# Bioenergetic failure, mitochondrial dysfunction and mitophagy

**DOI:** 10.1186/2193-1801-4-S1-L35

**Published:** 2015-06-12

**Authors:** Philip Beart

**Affiliations:** University of Melbourne, Australia

**Keywords:** Mitophagy, PINK1, autophagolysosome

Mitophagy specifically describes autophagy of damaged or dysfunctional mitochondria and occurs in programmed cell death when mitochondria fragment and remodel their cristae. Cellular bioenergetics is entwined with mitochondrial dynamics, and mitochondrial insults, including depolarization and inhibition of electron transport chain, trigger mitochondrial fragmentation. Here we investigated mitophagy in neurons during manipulation of mitochondrial bioenergetics. Dysfunction of mitochondria was induced by pharmacological inhibition of respiratory chain complexes I-V (rotenone, 3-nitropropionic acid, antimycin A, KCN & oligomycin, respectively) in primary cultures of cerebellar granule cells. The extent of bioenergetic failure was determined by measuring [ATP], depolarization of mitochondrial membrane potential and decrease in oxygen consumption rate in the Seahorse XF24. All stressors produced mitochondrial dysfunction as shown by concentration- and time-dependent decline in [ATP] over 4-24h. Complexes I, III or IV showed rapid loss of mitochondrial membrane potential and decreases in oxygen consumption rate over 4-24h. Autophagolysosomal flux was increased as shown by increased LC3-II and labelling of acidic vacuoles with monodansylcadaverine. Immunocytochemistry for PINK1 showed translocation of PINK1 from cytoplasm to mitochondria after injury, indicating likely involvement of mitophagy during bioenergetic dysfunction. Transfection of fluorescent pH-biosensor Rosella (Rosado CJ et al. Autophagy 4: 205 (2008)) targeting mitochondria indicated the pH of the mitochondrial location dropped with the inhibition of complex I and II, implying acidification of mitochondria, presumably in acidic vesicles undergoing mitophagy. Dieback of neuronal arbor observed here paralleled that seen with a GFP-plasmid. Together these data indicate that bioenergetic dysfunction produces mitophagy in primary neurons and is likely to be involved in neuronal dynamics.

